# D-Shaped Polarization Maintaining Fiber Sensor for Strain and Temperature Monitoring

**DOI:** 10.3390/s16091505

**Published:** 2016-09-15

**Authors:** Hummad Habib Qazi, Abu Bakar Mohammad, Harith Ahmad, Mohd Zamani Zulkifli

**Affiliations:** 1 Faculty of Electrical Engineering, Universiti Teknologi Malaysia, UTM, Skudai, 81310 Johor, Malaysia; hqhummad3@live.utm.my; 2 Photonics Research Centre, University of Malaya, 50603 Kuala Lumpur, Malaysia; harith@um.edu.my (H.A.); mohdzamani@um.edu.my (M.Z.Z.)

**Keywords:** fiber optic sensor, polarization-maintaining fiber, fiber loop mirror, strain sensor, temperature sensor, structural health monitoring

## Abstract

A D-shaped polarization-maintaining fiber (PMF) as fiber optic sensor for the simultaneous monitoring of strain and the surrounding temperature is presented. A mechanical end and edge polishing system with aluminum oxide polishing film is utilized to perform sequential polishing on one side (lengthwise) of the PMF in order to fabricate a D-shaped cross-section. Experimental results show that the proposed sensor has high sensitivity of 46 pm/µε and 130 pm/°C for strain and temperature, respectively, which is significantly higher than other recently reported work (mainly from 2013) related to fiber optic sensors. The easy fabrication method, high sensitivity, and good linearity make this sensing device applicable in various applications such as health monitoring and spatial analysis of engineering structures.

## 1. Introduction

Fiber optic sensor (FOS) technologies have gained wide interest for sensing applications during the recent decades due to comparative advantages over other conventional sensor technologies. These advantages include rapid responsiveness, compact size, safety, stability, flexibility, capability for remote monitoring and suitability to perform in harsh environments. FOS are designed to collect information via fiber optics, based on the fact that alterations in a specific physical property of a medium being sensed will cause a predictable change in the light transmission characteristics of the fiber. More recently, meaningful efforts have been made to develop an optical fiber–based sensing system for the simultaneous monitoring of multi-parameters, such as strain, temperature, vibration, refractive index and others [1,2,3,4,5,6,7]. In general, a fiber optic sensing system for simultaneous monitoring of multi-parameters requires a complex sensing head with multiple detection features, and each of them should exhibit a unique response to the external parameter to be measured [8]. It is not to say that all of these and other reported works are not capable of monitoring external parameters precisely; every contemporary sensing method possesses particular advantages and disadvantages. For example, Song et al. report a fiber Bragg grating (FBG)-based FOS for the simultaneous monitoring of strain and temperature sensing applications [9]. However, it has been observed that in the reported sensing device, the location of the FBG and the sensing element of the sensor was not same, which may cause the production of measurement errors in the sensing system. 

In principle, FOS rely on the fact that alterations in a specific physical property of a medium being sensed would cause a predictable change in the light transmission characteristics of the fiber [10,11,12]. To make an optical fiber sensitive to the external environment, the waveguide property is altered by some appropriate technique (generally by removing the cladding). Rather than removing the entire cladding of an optical fiber for a certain length, which causes the reduction of the strength, sustainability and durability, an alternative method is to use a D-shaped optical fiber sensor, which is comparatively strong and stable without compromising the sensitivity of the sensing device [13,14]. D-shaped optical fibers can be fabricated by removing a portion of cladding (lengthwise) of the optical fiber until the core of the fiber, and hence at a strong interaction between the evanescent field of the light signal and the external environment can occur at the polished surface [15]. Producing a D-sector on optical fibers through polishing techniques is appropriate [16]. Conventionally, an optical fiber is glued by epoxy in a cut curved V-shaped groove while its cladding is polished away over a limited interaction length of several millimeters. Depending upon the diameters of both the core and cladding, different sizes of V-shaped grooves have to be prepared precisely in advance by complicated techniques to produce a D-shaped sensor with desired dimensions [17]. Furthermore, this technique may suffer from fiber deformations such as scratches, cracks, or broken regions if the polishing protocol is not proper for the materials of the optical fiber and the surroundings. Thus, the polishing process needs very careful attention during the application. Beside all of these, the major drawback of the conventional method of the polishing technique is setting the fibers permanently into the V-shaped groove, which challenges the main advantages offered by optical fiber sensors, such as small size, easily access to hard-to-access areas, and of course the mobility of the sensing device. This makes the device less suitable from a practical point of view. 

Alternatively, a D-sector on an optical fiber can be produced by using a CO_2_ laser or femtosecond laser machining [18,19,20,21]. In general, plastic cladding can easily be stripped with a desired dimension to expose the fiber core by CO_2_ laser machining [22,23]. However, this technique is not useful to alter the silica-based optical fibers. Recently, Chen et al. introduced a new type of multi-D-shaped optical fiber sensor to monitor the refractive index of liquids [20]. In this work, the femtosecond laser pulse was used to fabricate a number of D-sectors on multimode optical fibers. However, the shorter length (1 mm) along the axis of the optical fiber makes this device less suitable for fiber optic–based sensing applications. Table 1 summarizes the currently reported D-shaped optical fiber sensors in the literature.

Furthermore, in optical fiber sensing applications where the external parameters do not have significant effects on the light transmission characteristics, i.e., attenuation, reflection, etc., and the fiber optic sensing system is unable to perform well, it is possible that the polarization of the optical wave may have a significant effect on the sensing system behavior and monitoring of the wavelength shift may give some meaningful information [28]. In such applications, utilization of single-mode fibers may suffer from unpredictable phase shift and/or time-varying polarization changes because of various external parameters and hence may cause errors in the measurements. The remedy of this problem is to introduce a polarization-maintaining fiber (PMF) in the sensing system [29].

To improve the above shortcomings, this paper presents a D-shaped PMF loop mirror-based FOS for the simultaneous monitoring of strain and the surrounding temperature. A mechanical end and edge polishing system with aluminum oxide polishing film was utilized to perform sequential polishing on one side (lengthwise) of the PMF in order to obtain a D-shaped cross-section in the laboratory environment. Adjusting the specific mechanical parameters allowed the control of the D-shaped zone. To meet the accuracy and repeatability requirements, optical power loss was monitored during the entire polishing process in situ and in real time. The performance of the D-shaped PMF sensor has been analyzed to monitor the applied strain and the variation in the surrounding temperature. Experimental results show that the proposed sensor has high sensitivity of 0.046 nm/µε and 0.13 nm/°C for strain and temperature, respectively. The sensor has a linear response over a measurable range of 0 to 50 µε for strain and 30 °C to 80 °C for temperature, respectively.

## 2. Fabrication Process

In this work, a new polishing technique is employed to produce D-sector on a PMF (PANDA, 1027-C, Yangtze Optical Fibre and Cable Joint Stock Limited Company (YOFC), Wuhan, China). An ULTRAPOL end and edge polishing system (model 3690.1) is the principal equipment utilized for the fabrication of a D-sector on the PMF. This particular polishing system is designed to polish ends and edges of waveguides and cannot be used to polish along the length of an optical fiber without mechanical alteration. The lengthwise polishing of PMF, to fabricate a D-sector on it, using this particular polishing system is possible after installing a custom die made by a cast acrylic sheet. The major advantages of using this polishing system are: it is comparatively simple, fast, safe, stable, and straightforward. Moreover, it allows a certain amount of flexibility in terms of the shape of the exposed area and also the thickness of the polished core.

**Figure 1:**
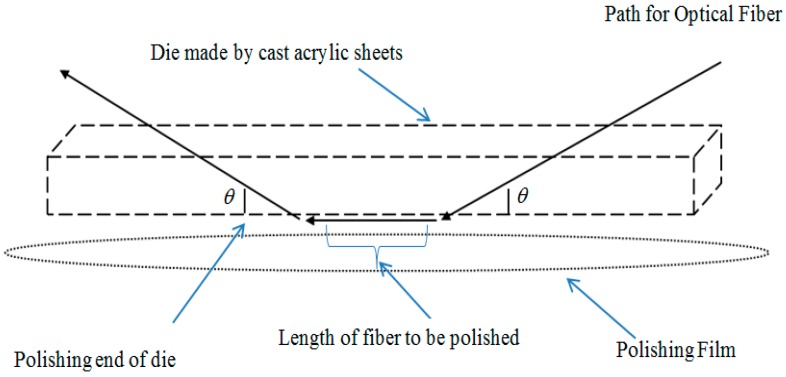
Conceptual illustration of the polishing system.

A conceptual illustration of the polishing system is given in Figure 1, where arrowed lines indicate the pathway of the optical fiber in the die and *θ* is the angle made by both ends of the optical fiber with the polishing end of the die. The rectangle shape with dashed outlines in the figure represents the acrylic sheet, while the round shape indicates the polishing film under the die. By monitoring the transmitted optical power loss and microscopic measurements of the D-sector during the polishing process, the D-sector is fabricated on the PMF with specific intended dimensions.

A cast acrylic sheet was used to build a base of 5 × 1 × 0.5 (l × w × h) cm^3^ dimensions for the polishing die. A holding strip in the die center allowed for a secure attachment of the die to the holder of the polishing system. Two tiny holes of 1 mm diameter are made in a vertical cross-section such that the distance between these two holes at the polishing surface was equal to the intended length for polishing. The angle *θ* between the polishing surface and the tiny holes was varied from 20° to 45° in order to determine the optimum efficiency for polishing. This polishing system can polish a waveguide surface maximum up to 8 in. When θ is less than 20°, the length of the polishing surface unnecessarily increases. Furthermore, fixing the die at the center of the polishing film causes it to crash the fiber all of sudden, because of the rotatory motion of polishing film under the polishing surface. On the other hand, through experiment it is found that θ greater than 35° causes an increase in the risk of fiber breakage during the polishing process and the consequent cost escalation of a single run. Experimental results from varying the angle indicate that θ ≈ 30° was the optimum angle for holes.

An optical fiber was threaded through both holes and tightly fastened with cellophane tape to the upper surface of the die to avoid misplacement during the polishing process. The die holding strip was next fixed securely in the holding arm of the polishing system. Appropriate polishing film was then applied at the polishing plate, the number of rotations per minute was set, and the timer was configured for an appropriate duration. The polishing system was subsequently engaged to start polishing one lengthwise side of the optical fiber so as to gradually fabricate a D-sector on the optical fiber.

Polishing on the PMF was performed using the polishing system with a 30 μm layer of aluminum oxide polishing film along with 90 polishing plate rotations per minute. To meet the accuracy and repeatability requirements, optical power loss was monitored during the entire polishing process in situ and in real time. This made it possible to fabricate a D-sector on optical fiber with a desired height more precisely and accurately. For the sake of validation, precision and repeatability of the proposed polishing technique, three sets of single-mode optical fibers (each set contains five PMFs) were polished until transmission power loss was reached at −3 (±0.04), −6 (±0.04) and −9 (±0.04) dB, respectively.

**Figure 2:**
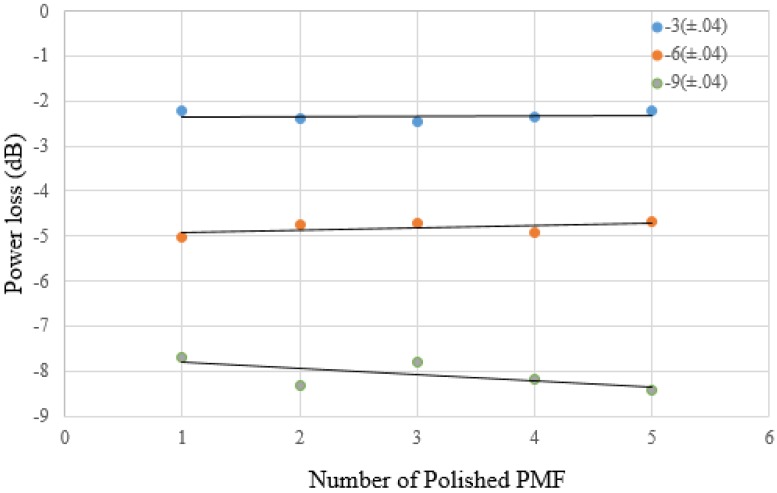
Power loss of each polished PMF (during and after the polishing process), three sets of PMFs (each set contains five optical fibers) were polished until transmission power loss was reached at −3 (±0.04), −6 (±0.04) and −9 (±0.04) dB, respectively.

**Figure 3:**
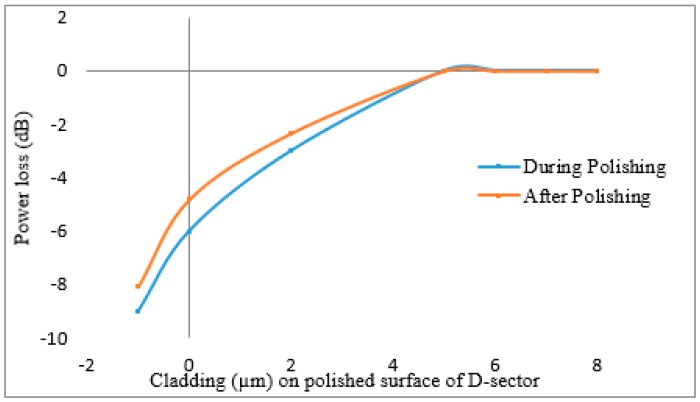
Average transmission power loss as a function of residual cladding on polished surface.

**Figure 4:**
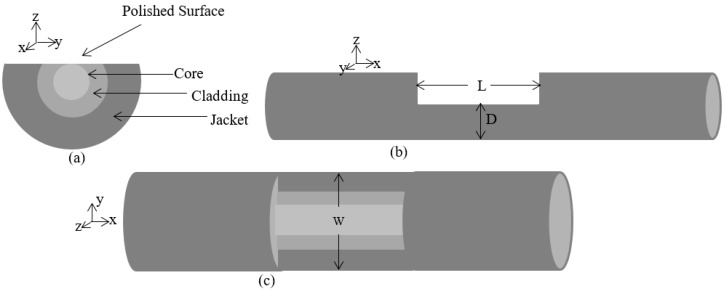
Conceptual illustration of D-sector on PMF: (**a**) Cross-sectional view at *Y*-*Z* plane, (**b**) cross-sectional view at *X*-*Z* plane and (**c**) cross-sectional view at *Y*-*X* plane; L is the length of the polished surface, D is the diameter of the D-sector and W is the width of the D-sector.

Figure 2 presents the power transmission loss of each of the polished optical fibers during and after the polishing process (while the optical fiber was unplugged from the polishing system). Figure 3 presents the average transmission power loss as a function of the residual cladding on the polished surface of the D-sector, during and after the polishing process. The conceptual illustration of the D-sector on the PMF is presented in Figure 4. 

## 3. Experimental Setup and Sensing Principle

**Figure 5:**
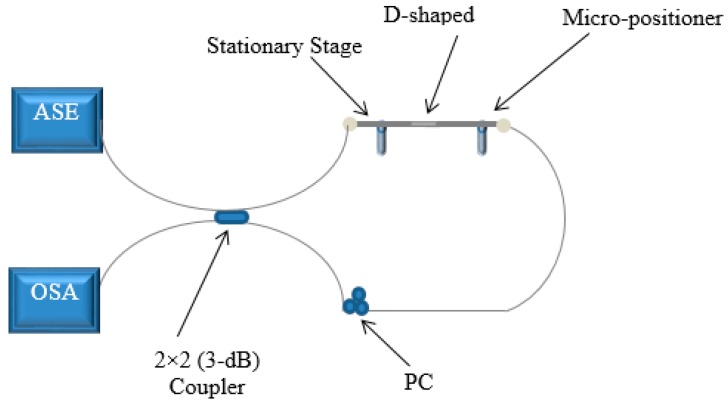
Schematic diagram of experimental setup of proposed strain and temperature sensor.

Refer to Figure 4, where L is the length, D is the diameter and W is the width of the D-sector. In this work a D-shaped PMF sensor (with dimensions of 10 mm × 205 µm × 195 µm (L × D × W)) is used to analyze the sensor’s performance to monitor the variation in the applied stress and the surrounding temperature. Before the experiment is conducted to analyze the sensor from a practical point of view, a power transmission loss of 2.46 dB is observed in the sensing device. A schematic diagram of the experimental setup for the proposed sensor is presented in Figure 5. In this work, an amplified spontaneous emission (ASE) source with a wavelength range of 1450–1650 nm is used as the light source. The light source is connected to the input port of the fiber loop mirror. The fiber loop mirror is constructed by splicing a D-shaped PMF sensor along with a polarization controller (PC) to a 2 × 2 3-dB coupler. The purpose of introducing PC in the sensing system is only to enhance the fringe visibility of the interference pattern. An optical spectrum analyzer (OSA, Yogogawa AQ6370C, Tokyo, Japan) connected at the output port of the fiber loop mirror, to complete the experimental set-up. The input light signal from the ASE is equally split and transmitted in both directions, clockwise and counter-clockwise in the fiber loop mirror (FLM). 

Due to the birefringence property of PMF, a relative phase difference is produced in the counter-propagating light beams. Interference is generated when both of the light beams are recombined at the 3 dB coupler. The optical transmission intensity $I_{T}$ in terms of phase difference can be defined as [24]:

(1) $$I_{T}=\frac{I_{ο}\left(1-\cos\varphi \right)}{2}$$

and

(2) $$\varphi =\frac{2\pi LB}{\lambda }$$

where φ is the phase difference, *λ* is the wavelength of interest for the light source, $I_{ο}$ is the intensity of the input light, L is the length of the PMF and $B=n_{s}-n_{f}$ is the birefringence index of the PMF. Further, $n_{s}$ and $n_{f}$ are the effective refractive indices of the slow and fast axis of the PMF, respectively. Also, the resonant dip wavelength satisfies the equation φ=2kπ and k is a random integer. So, the resonant dip wavelength can be expressed as:

(3) $$\lambda =\frac{LB}{k}$$

The changing of the external strain or temperature can cause a change of the length L and the birefringence B of the PMF. This characteristic leads to a wavelength shift of the interference spectra. The relationship between the wavelength shifts ∆λ and ∆B and ∆L (as strain ε=∆L/L) can be expressed as:

(4) $$∆\lambda =\;\frac{\left(L∆B+B∆L\right)}{k}=\frac{L}{k}\left(P_{t}∆T+B\varepsilon \right)$$

where $P_{t}$ is the thermo-optic constant of PMF, ∆L is the change in length of PMF caused by the applied strain and ∆B is the birefringence change caused by the variation of the surrounding temperature, while ∆T is the variation in the surrounding temperature [30].

It is clear that any change in the surrounding medium directly affects the propagation constants or the length of the D-type PMF loop mirror, which can be quantified either in terms of power loss or the spectral location of the peak/dip. In this work, two consecutive resonant dip wavelengths (*λ*_1_ and *λ*_2_) are chosen as measurement parameters for simultaneous monitoring of the strain and temperature.

## 4. Results and Discussion

**Figure 6:**
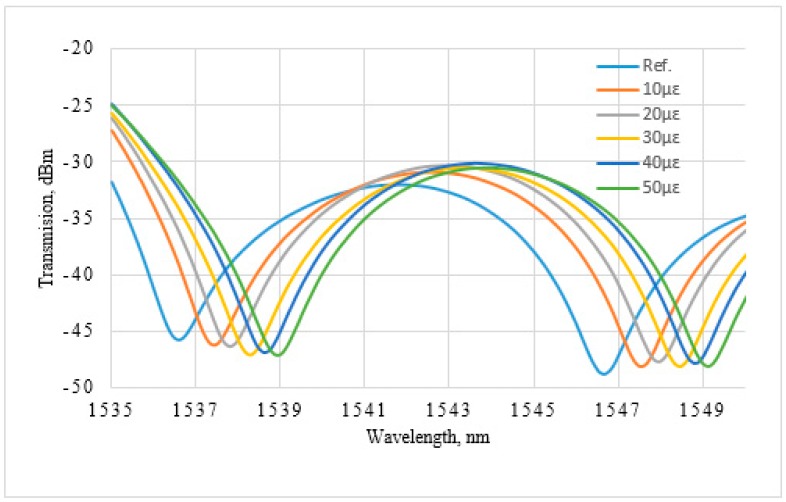
Spectral variation of D-type PMF loop mirror as a function of strain.

**Figure 7:**
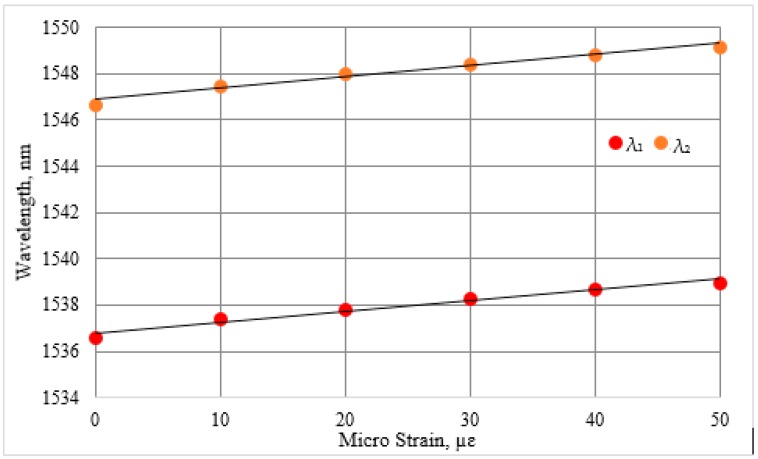
Wavelength shift of λ_1_ and λ_2_ as a function of strain.

**Figure 8:**
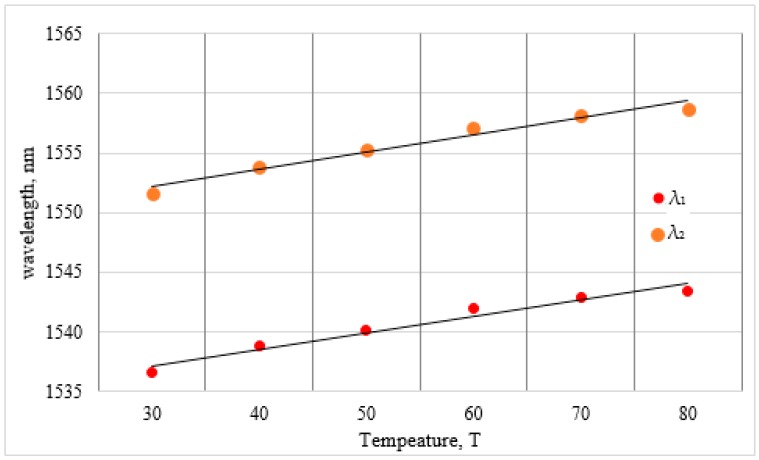
Wavelength shift of λ_1_ and λ_2_ as a function of temperature.

The performance of the proposed D-type PMF loop mirror sensor has been characterized for strain and temperature. In this work, strain and temperature are applied using a movable stage and a heating plate, respectively. Figure 6 presents the spectral variation of the D-type PMF loop mirror as a function of the strain, ranging from zero micro-strain (µε) to 50 µε. Two consecutive minima fringes at *λ*_1_ = 1536.6 nm and *λ*_2_ = 1546.7 nm were chosen to measure the spectral strain variation as a function of the strain. The relationships between the strain and the wavelength variation for both *λ*_1_ and *λ*_2_ are presented in Figure 7. A linear fit with a slope of 0.046 nm/µε and 0.047 nm/µε is obtained for both *λ*_1_ and *λ*_2_, respectively. To monitor spectral variation as a function of the temperature, two consecutive minima fringes at *λ*_1_ = 1536.58 nm and *λ*_2_ = 1551.65 nm at 30 °C were chosen as a reference. Figure 8 presents the relationship between the wavelength variation and the temperature for both of the wavelengths *λ*_1_ and *λ*_2_. The temperature sensitivity for the proposed sensor corresponding to *λ*_1_ and *λ*_2_ is 0.13 nm/°C and 0.14 nm/°C, respectively. The fitting linearity coefficients R^2^ for the applied strain, for both *λ*_1_ and *λ*_2_, are 0.977 and 0.973, respectively, as presented in Figure 7. The linearity coefficient changes in the surrounding temperature are 0.963 and 0.961 for both *λ*_1_ and *λ*_2_, respectively. The sensitivity coefficient of the reported sensor for both strain and temperature is given in Table 2.

From the aforementioned characterization of the D-shaped PMF loop mirror, it is clear that the D-type PMF sensor has a linear and independent response for strain and temperature. 

Therefore, it is possible to write two independent equations allowing explicit solutions for the actual value of each parameter, even in the situation when both parameters are changing. For this purpose, the matrix method can be used to determine the variation in both of the parameters for which the sensor is sensitive to [31]. Hence, the change in strain and surrounding temperature can be obtained simultaneously by using following matrix equation:

(5) $$\left[\begin{array}{c} \Delta T\\ \Delta \varepsilon \end{array}\right]=\frac{1}{D}\left[\begin{array}{cc} K_{\varepsilon 2} & -K_{\varepsilon 1}\\ -K_{T2} & K_{T1} \end{array}\right]\left[\begin{array}{c} \Delta \lambda_{1}\\ \Delta \lambda_{2} \end{array}\right]$$

where $D=K_{T1}K_{\varepsilon 2}-K_{\varepsilon 1}K_{T2}$, and the strain and temperature coefficients are obtained by fitting the experimental data using the linear regression as presented in Figure 7 and Figure 8. The above matrix system becomes:

(6) $$\left[\begin{array}{c} \Delta T\\ \Delta \varepsilon \end{array}\right]=-\frac{1}{0.003}\left[\begin{array}{cc} 0.047 & -0.046\\ -0.14 & 0.13 \end{array}\right]\left[\begin{array}{c} \Delta \lambda_{1}\\ \Delta \lambda_{2} \end{array}\right]$$

The performance of the sensor has been analyzed for monitoring the variation in the strain and surrounding temperature over a range of 50 µε and 50 °C, respectively. The maximum error was found to be ±0.003 nm/µε and ±0.01 nm/°C for the strain and temperature correspondingly, which is negligible in practical applications. 

## 5. Comparison of this Sensor with Other FOS for Strain and/or Temperature

Despite of all the progress in the field of fiber optic sensing techniques, to differentiate and analyze the simultaneous effects of various external parameters, such as strain, temperature, refractive index, pressure, etc., on fiber optic sensing systems is critical to the large-scale success of any fiber sensing technique. This is not only because the cross-sensitivity is a key issue for the practical applications of fiber optic sensors, but multi-parameter sensors can also reduce the complexity of the sensing systems [32]. Recently, Changyu Shen et al. presented a low-birefringence PMF-FLM interferometer temperature-insensitive strain sensor [30]. The proposed strain sensor was formed by inserting two equal sections of low-birefringence PMFs into a FLM. Two equal-length sections of PMFs were separated by a single-mode fiber (SMF). One of the PMFs was used as the sensing element, and the other one was used as the temperature compensation element. The sensitivity of the proposed sensor was 3.5 pm/με. In another work, a slightly tapered optical fiber with an inner air cavity is proposed and demonstrated for simultaneous strain and temperature measurements [33]. The sensing device was fabricated by using femtosecond laser micromachining together with fusion splicing techniques, followed by a short-duration tapering process. The reported sensor exhibited sensitivity of −16.12 pm/με and 85 pm/°C for both strain and temperature, respectively. Table 3 presents a comparative study in terms of sensitivity between the sensor presented in this work and more recent FOS works (mostly from 2013 to onward) for strain and/or temperature sensing applications. It is clear from the table that the reported sensor possesses significantly high sensitivity toward strain and temperature compared to its competitors. Moreover, a simple, rapid, safer and more stable fabrication method to fabricate this sensor and a straightforward monitoring procedure make this device more suitable from a practical point of view. 

## 6. Conclusions

A compact size D-shaped PMF loop mirror sensor for the simultaneous monitoring of strain and temperature has been presented and characterized successfully. A mechanical end and edge polishing system with aluminum oxide polishing film was utilized to perform sequential polishing on one side (lengthwise) of the PMF in order to obtain a D-shaped cross-section in the laboratory environment. Adjusting specific mechanical parameters allowed the control of the D-shaped zone. The major advantages of using said polishing system are that it is comparatively simple, fast, safe, stable, and straightforward. Moreover, it allows a certain amount of flexibility in terms of the shape of the exposed area and also the thickness of the core. To meet the accuracy and repeatability requirements, optical power loss was monitored during the entire polishing process in situ and in real time. Experimental results show that the proposed sensor has high sensitivity of 46 pm/µε and 130 pm/°C for variation in the strain and surrounding temperature, respectively, which is quite high compared to other recently reported (mainly from 2013) related fiber optic sensors. These values were found to have a linear response within the measureable range of 0 to 50 µε and 30 °C to 80 °C for strain and temperature, respectively. The easy fabrication method, high sensitivity, and good linearity would make this sensing device applicable in various applications such as health monitoring of spatial analysis of engineering structures. However, both minima (*λ*_1_ and *λ*_2_) possess nearly identical sensitivities for both strain and temperature (i.e., *Kε and K_T_*). Consequently, very careful measurements are required for both of the measuring variables (strain and temperature) for simultaneous monitoring purposes, as a small measurement error in both minima (*λ*_1_ and *λ*_2_) for both applied strain and temperature will be a cause for producing a significant error in the inversion calculations and hence will have a significant effect on the accuracy of the method. Authors are working to optimize the sensor’s performance so that the sensor will be able to perform over a large measurement range with high sensitivity and precision.

## Figures and Tables

**Table 1:** Summary of D-shaped optical fiber sensors.

No.	Type of Fiber	Polishing Technique	Dimension (h × w × l) *^1^	Application	Ref.
1	Multimode optical fiber	Femtosecond laser micromachining	100 µm × 250 µm × 1 mm	Liquid refractive index measurements	[20]
2	Polarization-maintaining fiber	Hydrofluoric acid etching	-	Liquid refractive index measurements	[24]
3	Plastic Optical Fiber	Rotating grinding wheel	10 mm (length)	Liquid refractive index measurements	[25]
4	Single-mode optical fiber	Femtosecond laser micromachining	60 μm × 75 μm (h × l)	Liquid refractive index measurements	[26]
5	Multimode optical fiber	Femtosecond laser micromachining	100 µm × 1 mm (h × l)	Liquid refractive index measurements	[21]
6	Single-mode optical fiber	Mechanical wheel lapping	4 mm (length)	Strain and Temperature	[27]

*^1^ h is the height of the D-sector, w and l are the width and length of the polished surface, respectively.

**Table 2:** Sensitivity coefficient of D-shaped PMF loop mirror for both strain and temperature.

	Kε (nm/µε)	K_T_ (nm/°C)
First Minima (λ_1_)	0.046	0.13
Second Minima (λ_2_)	0.047	0.14

**Table 3:** Comparison of the sensitivity of the sensor presented in this work with other recently reported (mainly from 2013) FOSs for strain and temperature.

No	Sensing Principle/Type of Fiber	Sensing Application	Sensitivity		Ref.
			Strain pm/με	Temperature pm/°C	
1	Hybrid optical fiber structure FLM-long period fiber gratings (LPFGs)-few mode fiber (FMF)	Strain and Temperature	−2.9	−17.6	[34]
2	FLM-low-birefringence PMF	Strain	3.5	-	[30]
3	No-core fiber (NCF)-fiber Bragg grating (FBG)-SMF	Strain and Temperature	1.19	12.8	[35]
4	Erbium-doped fiber (EDF)-FBG-Mach–Zehnder interferometer (MZI)	Temperature	-	18.8	[36]
5	Combined polymer and silica-based FBG	Strain and Temperature	1.48	-	[37]
6	Slightly tapered optical fiber with an inner air cavity	Strain and Temperature	−16.12	85.95	[38]
7	Dual gratings in one fiber	Strain and Temperature	2.57	8.5	[33]
8	PM-photonic crystal fiber (PM-PCF)	Strain	1.3	0.3	[39]
9	PM-PCF	Strain	2.04	-	[40]
10	PM-FLM	Strain and vibration	1.09	-	[3]
11	D-shaped PM-FLM	Strain and Temperature	46	130	This work
